# Exploration of the impact of multimode thermal therapy versus radiofrequency ablation on CD8^+^ T effector cells of liver malignancies based on single cell transcriptomics

**DOI:** 10.3389/fimmu.2023.1172362

**Published:** 2023-06-02

**Authors:** Ying Wang, Guang-Zhi Wang, Chao Chen, Hao-Zhe Huang, Yao-Hui Wang, Xin-Hong He, Lisa X. Xu, Li-Chao Xu, Wen-Tao Li

**Affiliations:** ^1^ Department of Interventional Radiology, Fudan University Shanghai Cancer Center, Shanghai, China; ^2^ Department of Medical Oncology, Fudan University Shanghai Cancer Center, Shanghai, China; ^3^ Med-X Research Institute, School of Biomedical Engineering, Shanghai Jiao Tong University, Shanghai, China; ^4^ Department of Medical Imaging Center, Affiliated Hospital, Weifang Medical University, Weifang, Shandong, China

**Keywords:** CD8^+^ effector T cells, liver cancer, radiofrequency ablation, multi-mode thermal therapy, single-cell RNA sequencing

## Abstract

**Introduction:**

Multimode thermal therapy (MTT) is an innovative interventional therapy developed for the treatment of liver malignancies. When compared to the conventional radiofrequency ablation (RFA), MTT typically offers improved prognosis for patients. However, the effect of MTT on the peripheral immune environment and the mechanisms underlying the enhanced prognosis have yet to be explored. The aim of this study was to further investigate the mechanisms responsible for the difference in prognosis between the two therapies.

**Methods:**

In this study, peripheral blood samples were collected from four patients treated with MTT and two patients treated with RFA for liver malignancies at different time points before and after the treatment. Single cell sequencing was performed on the blood samples to compare and analyze the activation pathways of peripheral immune cells following the MTT and RFA treatment.

**Results:**

There was no significant effect of either therapy on the composition of immune cells in peripheral blood. However, the differential gene expression and pathway enrichment analysis demonstrated enhanced activation of T cells in the MTT group compared to the RFA group. In particular, there was a remarkable increase in TNF-α signaling via NF-κB, as well as the expression of IFN-α and IFN-γ in the CD8^+^ effector T (CD8^+^ Teff) cells subpopulation, when compared to the RFA group. This may be related to the upregulation of PI3KR1 expression after MTT, which promotes the activation of PI3K-AKT-mTOR pathway.

**Conclusion:**

This study confirmed that MTT could more effectively activate peripheral CD8^+^ Teff cells in patients compared with RFA and promote the effector function, thus resulting in a better prognosis. These results provide a theoretical basis for the clinical application of MTT therapy.

## 1 Introduction

The liver is where primary cancer and metastases mostly occur. However, only few patients are considered suitable for hepatectomy or liver transplantation. Therefore, researchers have explored alternative treatment options for these patients. In the last decade, locoregional liver-directed therapies have been developed, with tumor ablation showing the most potential among them (1). Radiofrequency ablation (RFA) and cryoablation are commonly employed ablation techniques in clinical practice for localized tumor treatment. Both methods have been shown to be safe and effective (2, 3). Nevertheless, similar to other localized treatments, tumor recurrence and metastasis after RFA or cryoablation remains a significant obstacle in the treatment of hepatic malignancies (4, 5).

Multimode thermal therapy (MTT) is a novel treatment method consisting of continuous rapid freezing, natural thawing, and radiofrequency heating of the target tumor tissue (6). Our previous studies have shown (7, 8) that MTT has a good therapeutic effect on the 4T1 breast cancer mouse model generated by subcutaneous injection and the B16F10 melanoma lung metastasis mouse model. In addition, MTT can mediate the memory response of CD4^+^ T-helper type (Th) 1 (7, 8) and promote the release of heat shock protein 70 (Hsp70), maturation of dendritic cells (DCs) (8) and significant upregulation of interleukin (IL)-6 (9). A pliot study conducted by us also has shown that MTT induces functional maturation of DCs, promotes CD4^+^ T-cell-mediated anti-tumor responses, and reduces regulatory T cells (Tregs), thereby improving treatment outcomes in colorectal cancer patients (10). This study indicated that there is no notable difference in the proportion of CD8^+^ T cells between the two treatment methods, but MTT appeared to be superior in promoting IFN-γ expression in CD8^+^ T cells compared to RFA (10). CD8^+^ T cells are the primary population of antitumor effector cells. When circulating CD8^+^ T cells migrate and infiltrate tumor tissue, they encounter tumor antigens and become activated by the co-stimulation of antigen-presenting cells (APCs). These activated CD8^+^ T cells are then transformed into anti-tumor effector cells. However, few studies have compared the effects of MTT and RFA on CD8^+^ effector T (CD8^+^ Teff) cells at the single-cell level for the treatment of liver malignancies.

Single-cell transcriptomics are currently transforming understanding of cellular diversity and its function in health and disease. Researchers have started to use single cell transcriptomics to investigate key cellular and molecular functions in liver malignancies. Single-cell technology can improve understanding of immune cell heterogeneity and function in a variety of contexts, providing a new strategy for immunotherapy. It was found in previous studies that patients have a better prognosis after MTT treatment compared to RFA treatment (10). However, it is still unknown how the peripheral blood environment is changed and what is the mechanism responsible for the superior prognosis after MTT treatment. To investigate these inquiries, this study conducted single-cell RNA sequencing (scRNA-seq) on peripheral blood mononuclear cells (PBMCs) collected from six liver malignancies patients at different time points before and after MTT or RFA treatment. The aim was to compare the changes in the peripheral immune environment and provide comprehensive characterization of the immune response.

## 2 Materials and methods

### 2.1 Patients and ablation procedures

This clinical study was approved by the Ethics Committee of Fudan University Shanghai Cancer Center (No.2108241-11) and complied with the Declaration of Helsinki. All patients signed the informed consent.

In this study, blood samples were collected from six patients who received RFA or MTT at four different time points: pre-treatment (0d), three days after treatment (3d), seven days after treatment (7d), and 28 days after treatment (28d). All blood samples underwent cell isolation to get peripheral blood mononuclear cells (PBMCs) for single cell sequencing.

Previously, the three HCC patients and the one rectal cancer liver metastasis patient did not receive any systemic treatment, while the two pancreatic cancer patients were performed first-line treatment which contained gemcitabine at least four weeks before. All patients were not allowed to receive any systemic therapy within 28 days after treatment.

Of the six patients, two patients received RFA and four patients received MTT. RFA was performed using internally cooled electrode (19 gauge, MedSphere, Shanghai, China). A multimode monopole probe (17 gauge, MAaGI Medical Technology Co., Ltd, Shanghai, China), which was able to integrate freezing and thermal ablation, was used in MTT. Both RFA and MTT were performed under CT guidance (64-slice spiral CT; 120 kV, 250 mA, and 3-mm thickness; Philips Healthcare, Andover, MA, USA) by interventional radiologists with at least 5 years of experience in image-guided percutaneous ablation. The patient characteristics is presented in **Table 1**.

**Table 1:** Patient characteristics.

No.	Name	Sex	Age	Primary tumor	Group	Systemic therapy previously	Lesion number	Diameter (cm)
1	HCC1	M	54	HCC	RFA	no	1	2.29
2	HCC2	M	69	HCC	MTT	no	1	2.57
3	HCC3	M	69	HCC	MTT	no	1	1.49
4	PC1	M	55	pancreatic cancer	RFA	yes	1	2.62
5	PC2	F	48	pancreatic cancer	MTT	yes	2	1.94; 1.82
6	RC	F	76	rectal cancer	MTT	no	1	1.76

Enhanced MRI or CT was used for imaging evaluation after ablation. Local recurrence was defined as fulfilling at least one of three predefined morphological criteria on follow-up CT or MRI images (1): new tumor nodule within or adjacent to the ablative margin (2); clear rim of tissue abutting the ablation zone with different attenuation or enhancement than the ablation zone and adjacent normal hepatic parenchyma; and (3) at least 20% increase in the largest diameter of the ablation zone (11–13).

### 2.2 Isolation of PBMCs and single-cell RNA sequencing

Fresh PBMCs in the EDTA tube were transferred with the pipette with a filter core to a 15 ml low-adsorption centrifuge tube and centrifuged at 2000 rpm at room temperature for 10 min. The plasma layer was sucked (and stored at -80°C if needed). Suction was stopped when the plasma layer was about 0.5 cm from the blood cell layer. Blood cells were precipitated and re-suspended with phosphate-buffered saline (PBS) at a 1:1 ratio, and mixed thoroughly. The diluted blood cell suspension was dripped cautiously along the tube wall onto the surface of the equal amount of Ficoll-Paque Plus medium (GE Healthcare), and centrifuged at 2000 rpm at room temperature for 20 min, with the starting acceleration speed set at 9 and decelerating acceleration speed at 1. The buffy coat in the stratified solution was directly absorbed into a 15 ml centrifuge tube, added with 5 × volume PBS, and centrifuged at 1500 rpm at room temperature for 5 min, with both the starting and decelerating acceleration speeds set at 9. After discarding the supernatant, the obtained cell precipitation was blown gently with Ca/Mg-free PBS, re-suspended into a 1ml suspension, added with 2ml GEXSCOPE^®^ red blood cell lysis buffer (RCLB, Singleron), cultivated at room temperature or 25°C for 8~10 min, and centrifuged at 1300 rpm for 5 min. After discarding the supernatant, cells were re-suspended with 1ml PBS, precipitated, and counted. Trypan Blue Staining Solution was mixed with 10 μL PBMC suspension at a 1:1 ratio, from which 10 μL solution was sucked for cell counting, and at the same time the cell concentration and activity were recorded.

### 2.3 RT-PCR amplification and library construction

After discarding the supernatant, the remaining sample was re-suspended with PBS (HyClone) into 2×10^5^ cells/mL cell suspension, which was then added into the microwell chip using Singleron Matrix^®^ Single Cell Processing System. Using the same system, Barcoding Beads with barcode labeling were added to the microwell chip. After cytolysis, the polyT structure at the barcode end of the bead surface was coupled with the mRNA tail polyA sequence to capture mRNA. After the complete combination, the magnetic beads were harvested for cDNA reverse transcription and PCR amplification. The obtained cDNA through amplification was fragmented and connected with the sequencing adaptor. The scRNA-seq library was constructed by using GEXSCOPE^®^ Single Cell RNA Library Kits (Singleron) according to the manufacturer’s protocol (14). The single library was diluted to 4 nM, pooled, and sequenced with Illumina novaseq 6000, and the paired-end read was 150 bp.

### 2.4 Primary analysis of raw read data

The original readings from scRNA-seq were treated with CeleScope (https://github.com/singleron-RD/CeleScope) v1.5.2 to produce the gene expression matrix. Briefly, the original readings were first treated with CeleScope, and the low-quality sequences were removed with Cutadapt v1.17 (15) to line up the poly-A tail with the sequencing adaptor to extract the cell barcode and UMI. Subsequently, the sequencing text was compared with human reference genome GRCh38 by STAR v2.6.1a (16). The UMI count and gene expression count of each cell were obtained by featuCounts v2.0.1 (17), which were used to produce an expression matrix file for subsequent analysis.

### 2.5 Quality control, dimension reduction and clustering

Cells with fewer than 200 genes and those with the gene number and UMI in the top 2 percentages were filtered out. At the same time, cells containing more than 20% mitochondrial gene were also filtered out. After filtration, each sample containing a mean of 10000 cells was used for downstream analysis. Averagely, each cell had 1700 genes and 4983 UMIs. Using Seurat v3.1.2 function package (18), combined dimension reduction and cell clustering were performed. Then, using NormalizeData and ScaleData function, all gene expressions were normalized and scaled, and then using FindVariableFeautres function, the top 2000 variable genes were screened out for PCA analysis. Based on the top 20 main components, cells were categorized into several subsets by using FindClusters. Finally, cells in 2-dimensional space were visualized using UMAP algorithm.

### 2.6 Analysis of differentially expressed genes

To identify differential expression genes (DEGs), default parameters from Seurat FindMarkers function based on Wilcox Likelihood Ratio Test were used to select genes that expressed in more than 10% cells in the cluster and whose fold change was larger than 0.25 as DEGs. The cell type of each cluster was explained by typical marker genes of cells in combination with literature searching. Related gene expressions were identified in DEGs, and the expression of marker genes of various cell types was displayed by the heat chart/bubble chart/violin chart produced by the Seurat DoHeatmap/DotPlot/Vlnplot function. Two cells that contained the marker genes of different cell types were identified and deleted manually.

### 2.7 Cell type annotation

Using SynEcoSys databank, the type of each cell cluster was determined by the expression of the typical marker genes identified in DEGs. The heat chart/bubble chart/violin chart displaying the expression of these marker genes identified from the type of each cell were mapped out using Seurat v3.1.2 DoHeatmap/DotPlot/Vlnplot.

### 2.8 Expression pattern cluster

We used Mfuzz v2.46.0 (19) to identify time dependent transcriptional program in T cells. The samples were grouped according to the treatment time. First, the average expression of each gene was calculated for each stage. Next, over 25 percent of the genes that were assessed as absent were excluded. Replace the remaining deletion values with the mean expression values of the corresponding genes. Then, the “filter.std (min.std=0)” and “standardize ()” functions were performed for preprocessing according to the tutorial. After that, we clustered the genes into 6 expression patterns.

### 2.9 Pathway enrichment analysis

To investigate the potential function of DEGs, “clusterProfiler” R package v4.0.2 in combination with GO and KEGG databank was used for pathway enrichment analysis (20). Pathways with a P_adj value 0.05 were considered significantly enriched. Gene sets were classified as three categories: molecular function (MF), biological process (BP) and cell component (CC).

### 2.10 scGSVA

Single-cell data were analyzed by scGSVA. The pathway enrichment scoring matrix was generated by SSGSSA dada package (https://github.com/guokai8/scGSVA), which scores each cell by the SSGSEA method. Differential enrichment scores between different cell types were calculated by Limma package, and the absolute t value >1.96 was considered significant difference.

### 2.11 Trajectory analysis

Cell differentiation trajectories were reconstructed by Monocle2 v2.22.0 (21) and BEAM model (branching representation analysis model. Cell spatiotemporal differentiation sequencing was performed by highly variable genes (HVGs). FindVairableFeatures and dimensional reduction were performed by DDRTree. Finally, the trajectories were visualized by plot_cell_trajectory function.

### 2.12 Functional gene module analysis

Hotspot was used for identifying functional gene modules that account for heterogeneity in CD8^+^ Teff cells subgroups (22). In brief, it used the “danb” model to select the top 500 genes with the highest autocorrelation zscore to identify modules by create_module functions, where min_gene_threshold =15 and fdr_threshold = 0.05. The module score was calculated by module_score functions

### 2.13 Jaccard similarity analysis

Using the marker genes of CD8^+^Teff cells subsets, transcriptional similarity between the cell subsets was calculated by Jaccard similarity co-efficient function. Transcriptional similarity between 12 (23) CD8^+^Teff cell subsets and five cell types/statuses was analyzed by using the Jaccard similarity co-efficient of the top 50 marker genes.

### 2.14 Transcription factor regulatory network analysis (pySCENIC)

Transcription factor network was constructed by pyscenic (v0.11.0) (24) using scRNA expression matrix and transcription factors in AnimalTFDB. First, GRNBoost2 predicted a regulatory network based on the co-expression of regulators and targets. CisTarget was then applied to exclude indirect targets and search for transcription factor binding motifs. After that, AUCell was used to quantitate the regulon activity of each cell. According to Regulon Specificity Scores (RSS), cluster-specific TF regulons were identified and their activities were visualized in heatmaps.

### 2.15 UCell gene set scoring

Gene set scoring was performed using the R package Ucell v 1.1.0 (25). Ucell scores were based on the Mann-Whitney U statistic by ranking query genes in order of their expression levels in individual cells. As Ucell is a rank-based scoring method, it is suitable to be used in large datasets containing multiple samples and batches.

### 2.16 Statistics and repeatability

Comparison of cell distribution between two cell groups was verified by non-paired t test. Comparison of gene expression or signature between two cell groups was verified by non-paired two-tailed Wilcoxon rank sum test. Comparison of cell distribution between paired group 1 and 2 was verified by pared two-tailed Student’s t test. All statistical analyses and expressions were expressed in R language. All statistical tests used in the figures and tables are expressed in the legends. *P* < 0.05 was considered statistically significant. The exact value of n is expressed in the figures, tables and legends. The connotation of n is also expressed in the figures, tables and legends.

## 3 Result

### 3.1 Patient follow-up

Of the six patients, two lesions in RFA group and five lesions in MTT group were performed treatment. The follow-up lasted for 7-13 months. During the follow-up, one lesion in RFA group was observed with local recurrence, meanwhile, there was no local recurrence in MTT group. On the whole, the pancreatic cancer patients had the worst prognosis. The PC1 and PC2 patient died at 7-months and 10-months post-treatment, respectively, and new focus appeared at 7-month and 10-month post-treatment, respectively. The other patients remained alive. Of HCC and rectal cancer liver metastasis patients, only one HCC in RFA group appeared new focus in 11-months post-treatment.

### 3.2 Peripheral immune landscape in MTT-treated and RFA-treated patients

**Figure 1:**
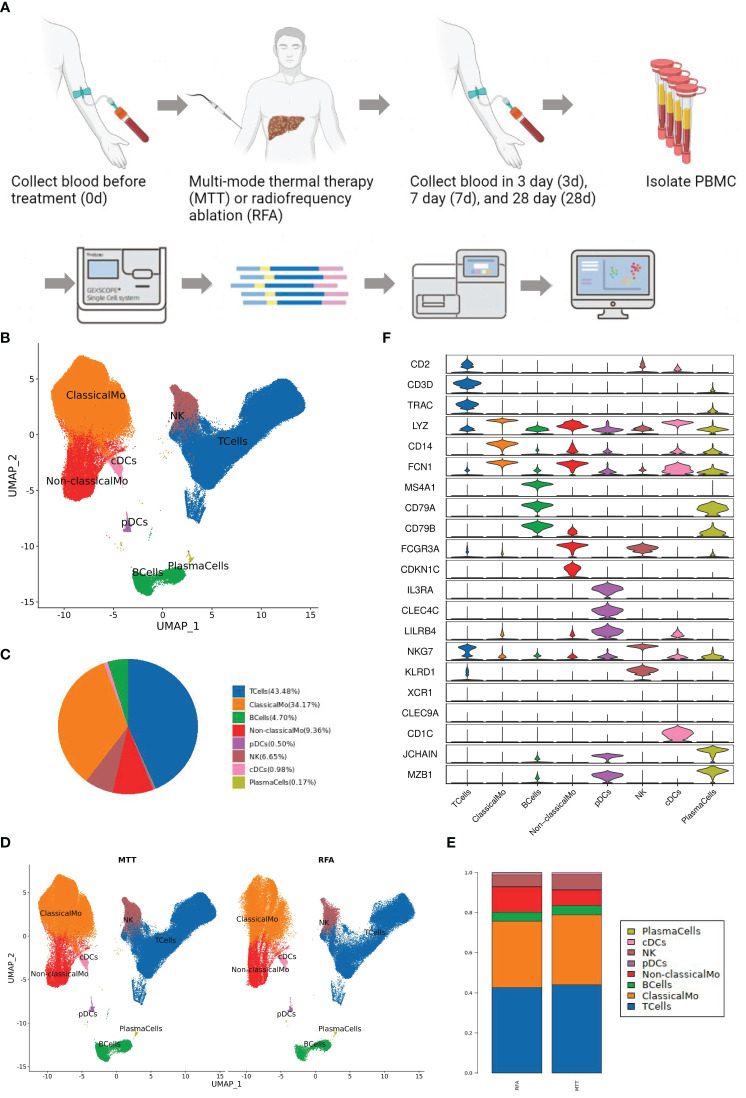
Peripheral immune landscape after MTT and RFA treatment. **(A)** Research design and workflow diagram (Created with BioRender.com). **(B)** Color the UMAP by cell type. Nine cell types were collected by clustering analysis, including T cells, NK, B cells, Plasma cells, Classical monocytes, Non-classical monocytes, cDC1, cDC2, and pDCs. **(C)** The pie chart showed that T cells and ClassicalMo accounted for 43.48% and 34.17%. B cells, Non-classicalMo, and NK accounted for 4.70%, 9.36%, and 6.65%. Other cells accounted for less than 1%. **(D)** Color the UMAP by group. **(E)** The histogram of cell proportion showed that the proportion of T and NK cells increased after MTT treatment. **(F)** Stacked violin diagram of the marker gene expression levels of different cell types.

To investigate the changes in the peripheral immune environment of patients treated with MTT and RFA, single-cell transcriptome sequencing was carried out on PBMC samples collected from 4 patients in the MTT group (MTT, N = 4) and 2 patients in the RFA group (RFA, N = 2) at 4 time points: pre-treatment (0d), short-term (3d) post-treatment, mid-term (7d) post-treatment, and long-term (28d) post-treatment, respectively. A single-cell atlas consisting of 23 PBMC samples was constructed (**Figure 1A**). First, a clustering analysis was performed using dimensionality reduction algorithm UMAP on all cells, and a total of 9 cell types were identified (**Figure 1B**), including T cells (CD2, CD3D, TRAC), NK (FCGR3A, KLRD1, NKG7), B cells (CD79A, CD79B, MS4A1), Plasma cells (CD79A, JCHAIN, MZB1), Classical monocytes (ClassicalMo, LYZ, CD14, FCN1, VCAN), Non-classical monocytes (Non-classicalMo, FCGR3A, CDKN1C), Conventional dendritic cells (XCR1, CLEC9A, FCER1A), and Plasmacytoid dendritic cells (pDCs, CLEC4C, IL3RA, LILRB4) (**Figure 1F**). Among them, T cells and ClassicalMo constituted the highest proportions, at 43.48% and 34.17%, respectively, followed by Non-classicalMo, NK, and B cells, at 9.36%, 6.65%, and 4.70%, respectively, while the proportions of other cells were less than 1% (**Figure 1C**).

Subsequently, the UMAP cluster plots were color-coded by group, and it was found that the distribution of immune cells was consistent between MTT and RFA groups (**Figure 1D**). Although the change in the ratio of immune cells was not signification in both groups, an increasing trend was observed in the proportions of T cells in the MTT group compared to the RFA group (**Figure 1E**). As mentioned above, ClassicaMo had a large proportion of the sample, up to 34.17%, however, the results of GSEA analysis showed that the regulation of antigen processing and presentation signaling pathway in ClassicalMo were significantly different between MTT and RFA group on day 3, while there were no significant differences on day 7 and day 28 (**Supplementary Figure S1**). Combining the above results and the previous studies, therefore, we, focus on T cells, to further investigate the differences in the characteristics of the peripheral immune environment and the mechanisms responsible for the superior prognosis of MTT over RFA.

### 3.3 Differences in immune function of peripheral T cells in MTT group and RFA group

**Figure 2:**
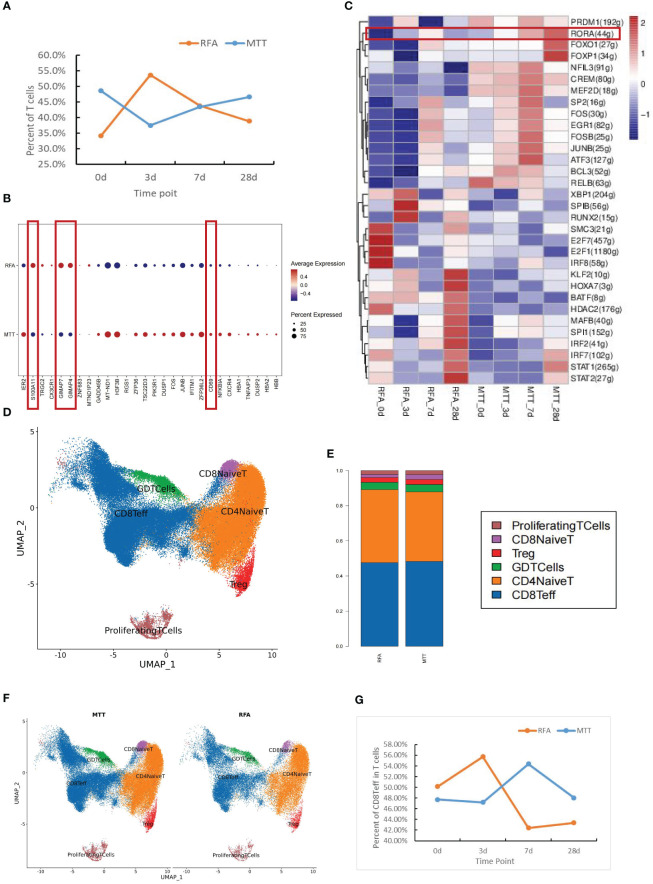
Differences in immune function of peripheral T cells in MTT and RFA treatment. **(A)** T cell proportion curve showed that the T cell proportion in MTT group was lower than that in RFA group at the early stage (3d) after treatment, but the T cell proportion in MTT group subsequently increased and was higher than that in RFA group at the late stage (28d). Blue represented the MTT (n=4) group and orange represented the RFA group (n=2). **(B)** Differentially expressed genes analysis of T cells showed that compared with RFA group, the expressions of *S100A11*, *GIMAP7*, and *GIMAP4* in MTT group (n=4) were decreased, while the expressions of CD69 were up-regulated. **(C)** Transcription factor analysis showed that RORA was upregulated over time after MTT treatment. **(D)** Six T cell subsets were obtained by UMAP cluster analysis. **(E)** The proportion of each cell subtype was shown by group. **(F)** Display the UMAP cluster diagram by group. **(G)** Changes in the proportion of CD8Teff in T cells after MTT and RFA.

T cells are the main component of lymphocytes, also known as T lymphocytes. They can directly kill target cells, regulate the production of antibodies by B cells in a stimulatory or inhibitory manner, respond to specific antigens and mitogens, and produce cytokines to protect against disease infection and tumor formation (26). As mentioned above, there was an increasing trend in the proportion of T cells relative to total cells after MTT treatment. To investigate the changes in T cell ratios, further analysis of the cell proportions in the two groups at different time points was conducted. The proportion of T cells in the MTT group was lower than that in the RFA group during the early post-treatment period (3d) but was increased during the mid-late post-treatment period (7d) and higher than that in the RFA group during the late post-treatment period (28d) (**Figure 2A**), possibly suggesting that MTT is more effective in maintaining peripheral T-cell abundance for a long period than RFA in patients with malignancy after treatment.

Previous studies have validated the efficacy of multimode thermal therapy in the treatment of colorectal liver metastases (CRCLM) and demonstrated that MTT may achieve a better prognosis than RFA by modulating CD4^+^ T cell function (10). To address how MTT regulates T cells at the transcriptional level to exert good anti-tumor effects, DEGs analysis was first performed on T cells. It was found that the expression of apoptosis-related genes of *S100A11*, *GIMAP7*, and *GIMAP4* was decreased and T cell activation marker CD69 was upregulated in the MTT group compared to the RFA group (**Figure 2B**). This suggested a decrease in the number of T cells after RFA treatment may be attributed to T cell apoptosis. In contrast, the expression of *GIMAP7* and *GIMAP4* was decreased after MTT treatment, which might lead to a relatively lower rate of T cells apoptosis and enhance their effector functions. The expression of CD69 was upregulated in T cells after MTT, which further indicated enhanced T cell activation compared to RFA treatment. Transcription factor analysis was also performed for both therapies, which revealed that the expression of transcription factor RORA was gradually increased from day 0 to day 28 after MTT treatment (**Figure 2C**). Thus, MTT treatment may promote T-cell activation and proliferation, which ultimately enhances effector functions, indicating a positive prognosis.

**Figure 3:**
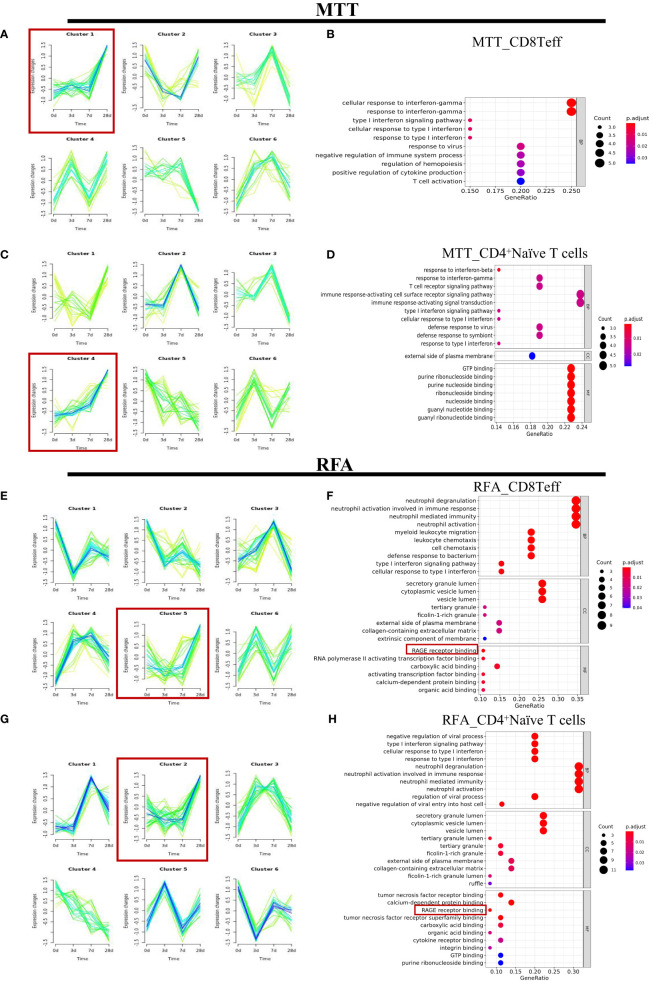
The expression patterns of CD8^+^Teff cells and CD4^+^ Naive T cells in the MTT and RFA group. **(A)** The expression patterns were performed for CD8^+^Teff of MTT group. **(B)** GO enrichment analysis was performed for fitting gene set cluster2 in CD8^+^Teff. **(C)** The expression patterns were performed for CD4^+^Naïve T cells of MTT group. **(D)** GO enrichment analysis was performed for fitting gene set cluster4 fitting gene set in CD4^+^Naive T cells. **(E)** The expression patterns were performed for CD8^+^Teff of RFA group. **(F)** GO enrichment analysis was performed for fitting gene set cluster2 in CD8^+^Teff of RFA group. **(G)** The expression patterns were performed for CD4^+^Naïve T cells of RFA group. **(H)** GO enrichment analysis was performed for fitting gene set cluster4 fitting gene set in CD4 Naive T cells.

To better define which subsets of T cells play a significant role in the therapeutic effect of MTT, the subsets were sorted and annotated, and a total of six different T cell subsets were identified, including CD8^+^ Teff cells, CD8^+^ naïve T cells, Treg, CD4^+^ naïve T cells, γδ T cells, and proliferation state T cells (**Figure 2D**). The distribution and proportions of T cell subsets were approximately the same for both therapies (**Figures 2E, F**). Expression pattern cluster was performed on six T cell subsets, revealing a significant upregulation trend of CD8^+^ Teff cells in cluster1, CD8^+^ naïve T cells in cluster6, Treg in cluster3, CD4^+^ naïve T cells in cluster4, γδ T cells in cluster4, Proliferation T cells in clusters 3 and 4 from days 0 to 28 in the MTT group (**Figures 3A, C**, **S2**). Subsequently, GO enrichment analysis was performed on these gene clusters showing upregulation trends. The majority of these clusters were enriched to T cell activation, type I interferon, antigen presentation, and other related immune signaling pathways, suggesting that various T cell subsets exerted immune killing functions in MTT treatment (**Figures 3B, D**, **S2**). In the RFA group, there was a significant upregulation trend of CD8^+^ Teff cells in cluster5, CD8^+^ naïve T cells in cluster6, Treg in cluster5, CD4^+^ naïveT cells in cluster2, γδ T cells in cluster2, and Proliferation T cells in cluster1 from day 0 to 28 (**Figures 3E, G**, **S3**). The GO enrichment analysis for CD8^+^ Teff cells in cluster6, CD8^+^ naïve T cells in cluster6, CD4^+^ naïve T cellsin cluster2, and γδ T cells in cluster2 all showed enrichment in the RAGE signaling pathway (**Figures 3F, H**, **S3**). RAGE is a multi-ligand pattern recognition receptor involved in various chronic inflammatory states. Under normal physiological conditions, the expression level of RAGE is low. However, it is highly upregulated under chronic inflammatory conditions due to the accumulation of various RAGE ligands. Therefore, the upregulation of Th17 after RFA treatment may contribute to a short PFS. The high expression of RAGE after RFA treatment may promote Th17 differentiation and, in turn, compromise postoperative outcomes.

### 3.4 PI3K-AKT-mTOR in CD8^+^Teff cells enabled immune function after MTT treatment

**Figure 4:**
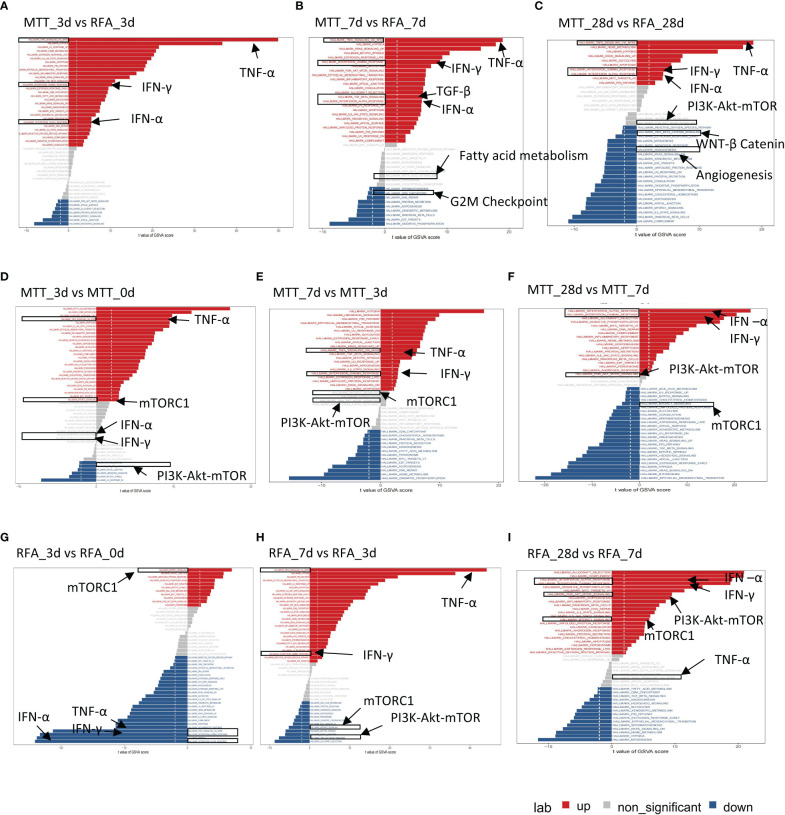
scGSVA analyzed hallmark signaling pathways at different times in RFA and MTT groups. **(A–C)** scGSVA analysis performed between MTT and RFA group at same timepoint. **(D–F)** scGSVA analysis performed between different time points in MTT group. **(G–I)** scGSVA analysis performed between different time points in RFA fgroup. Red represented the significant up-regulated pathway, blue represented the significant down-regulated pathway, and grey represent the non-significant up or down-regulated pathway.

**Figure 5:**
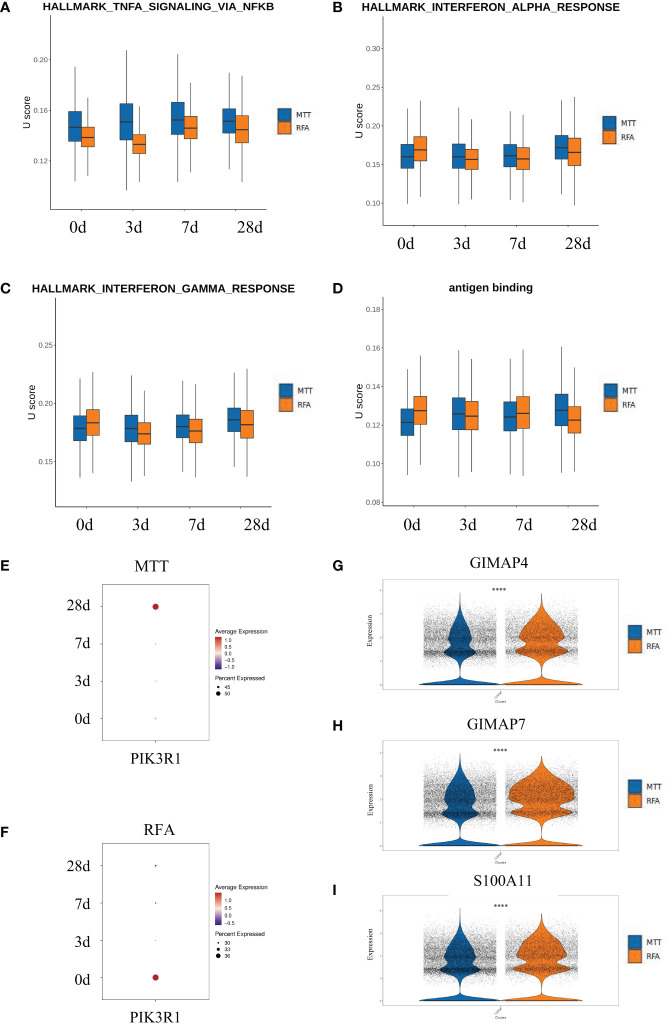
PI3K-AKT-mTOR in CD8^+^ Teff might enabled immune function after MTT treatment. **(A–D)** Four signaling pathways were analyzed by U cell score. Figure **(E, F)** Bubble map of pik3r1 over time after MTT and RFA treatment. **(G–I)** The violin diagram shows the expressions of S100A11, GIMAP7 and GIMAP4 in the MTT and RFA group. **** represents p<0.0001.

There was no significant difference in proportions of CD8^+^ Teff cells as well as the other five T cell subsets between the MTT and RFA groups. However, CD8^+^ Teff cells ratio was found to be increased in the MTT group and decreased in the RFA group when comparing the results on day 0 and day 28 (**Figure 2G**). Additionally, previous studies have reported that CD8^+^Teff cells play crucial roles in combating pathogenic injury and immune surveillance. To further clarify the mechanism responsible for the superior prognosis of MTT over RFA, an in-depth investigation was conducted on the difference in CD8^+^ Teff cells function between the MTT and RFA groups. scGSVA analysis was performed on CD8^+^ Teff cells in the two groups at different time points (**Figures 4A–C**). The results showed that on day 3, the MTT group had significantly upregulated TNF-α signaling pathway and IFN-γ response pathway, both of which are involved in immune function, compared to the RFA group. At the same time, there were also upregulated signals associated with immune suppression, such as apoptosis and MYC signaling pathways (**Figure 4A**). On day 7, the MTT group showed upregulation of immune function signaling pathways such as TNF-α, IFN-α , IFN-γ, and TGF-β, compared to the RFA group. Conversely, there was downregulation of signaling pathways associated with immune suppression, such as G2M_CHECKPOINT and fatty acid synthesis. On day 28, the MTT group showed upregulation of immune function signaling pathways such as TNF-α, IFN-α and IFN-γ, compared to the RFA group. Conversely, there was downregulation of signaling pathways associated with immune suppression, such as WNT-β, angiogenesis, and fatty acid synthesis signaling pathways. From day 3 to day 28, the immune function signaling pathway TNF-α remained significantly upregulated. To clarify the upregulation of inflammatory signals associated with MTT treatment, UCell analysis was carried out on HALLMARK_TNFA_SIGNALING_VIA_NFKB, HALLMARK_INTERFERON_ALPHA_RESPONSEH, HALLMARK_INTERFERON_GAMMA_RESPONSE, and antigen binding (**Figures 5A–D**). The results revealed that HALLMARK_TNFA_SIGNALING_VIA_NFKB scores were upregulated from day 0 to 28 (**Figure 5A**), and UCell scores of 3 remaining signaling pathways were upregulated from day 3 to 28 in the MTT group compared to the RFA group (**Figures 5B–D**). Subsequently, scGSVA analysis was conducted on day 3 vs day 0, day 7 vs day 3, as well as day 28 vs day 7 for both MTT and RFA groups. The results showed that in the MTT group, the mTORC1 signaling pathway was upregulated on day 3 vs day 0 and day 7 vs day 3, and downregulated in day 28 vs day 7. However, the PI3K-AKT-mTOR signaling pathway was upregulated on both day 28 vs day 7 and day 7 vs day 3, despite the downregulation of mTORC1 pathway (**Figures 4D–F**). In contrast, the mTORC1 signaling pathway was significantly upregulated on both day 3 vs day 0 and day 28 vs day 7 in the RFA group (**Figures 4G–I**). Further investigation was conducted to analyze changes in PIK3R1 expression over time in both treatment groups. The analysis revealed an increase in PIK3R1 expression over time in the MTT group (**Figure 5E**), while a decrease in expression was observed in the RFA group (**Figure 5F**). Meanwhile, inter-group difference analysis was performed on CD8^+^ Teff cells to further investigate the upregulation of the expression of tumor suppressor *S100A11* and apoptosis-related genes *GIMAP7* and *GIMAP4* in T cells. The results showed that the expression of *S100A11*, *GIMAP7*, and *GIMAP4* was decreased in the MTT group compared to the RFA group (**Figures 5G–I**). In summary, these results suggest that the CD8^+^ Teff cells-dependent PI3K-AKT-mTOR pathway regulates the cell cycle and reduces apoptosis after MTT treatment. This regulation promotes the expression of relevant inflammatory signals and exert a more significant function of immune regulation, which may contribute to the good prognosis associated with MTT treatment.

### 3.5 MTT treatment relied on CD8 Teff_3 to activate more CD8^+^Teff cells to exert immune function

**Figure 6:**
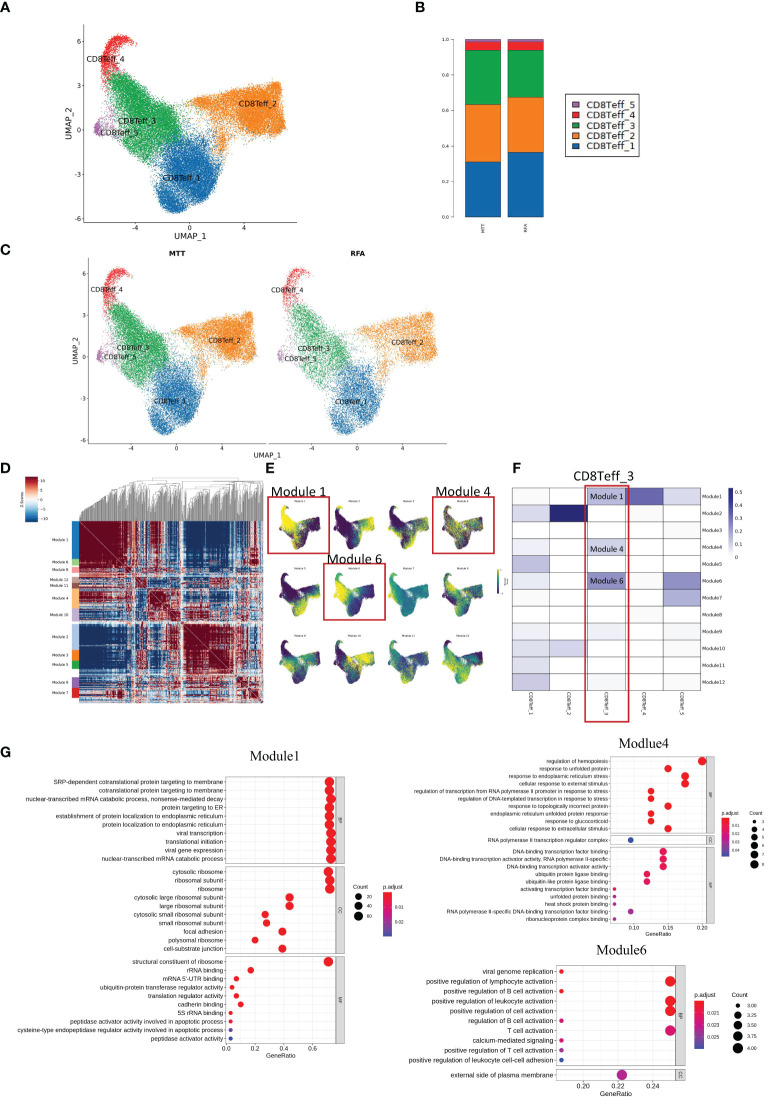
MTT treatment relied on CD8 Teff_3 to activate more CD8Teff to exert immune function. **(A)** UMAP showing five subgroups, namely CD8Teff_1, CD8Teff_2, CD8Teff_3, CD8Teff_4, and CD8Teff_5. **(B)** Display UMAP maps in groups. **(C)** Compared with RFA group, CD8Teff_3 in MTT group showed an obvious upward trend, while CD8Teff_1 showed a downward trend. **(D)** Hotspot analysis was performed on 5 subpopulations of CD8Teff cells and a total of 12 gene sets were fitted. **(E)** The gene set score reduction map showed that CD8Teff_3 was mainly module 1 and module 6. **(F)** Jaccard similarity analysis showed that CD8Teff_3 is mainly module 1, 4, and 6. **(G)** GO enrichment analysis was carried out for module 1, 4, and module 6.

To get more insight into how MTT relies on CD8^+^ Teff cells to exert strong immune functions, the heterogeneity of CD8^+^ Teff cells was investigated. First, unsupervised clustering analysis of CD8^+^ Teff cells was performed, and a total of five cell subsets were obtained, including CD8Teff_1, CD8Teff_2, CD8Teff_3, CD8Teff_4, and CD8Teff_5, with clusters 1, 2, and 3 having the highest proportions (**Figures 6A, B**). Then, the inter-group comparison was carried out on the 5 cell subsets, which revealed a significant increasing trend of CD8Teff_3 and a decreasing trend of CD8Teff_1 in the MTT group compared to the RFA group (**Figures 6B, C**). Subsequently, hotspot analysis of the 5 subsets was performed and a total of 12 gene sets were fitted (**Figure 6D**), which showed that CD8Teff_3 was mainly characterized by modules 1, 4 and 6 (**Figure 6E**). Further results of Jaccard similarity co-efficient function also showed that CD8Teff_3 had the highest similarity to modules 1, 4, and 6, while CD8Teff_1 did not have a clear association with specific modules (**Figure 6F**). Therefore, GO enrichment analysis was conducted mainly for the gene modules of CD8Teff_3. The results showed that modules 1 and 4 were associated with signals such as DNA and mRNA transcription, cellular response to external stimuli, and ribosome function, while module 6 was associated with signals such as T cell activation and differentiation (**Figure 6G**). Among them, CD8Teff_3 showed the highest similarity with module 6 (**Figure 6F**), suggesting that CD8Teff_3 might be closely related to T cell activation-related functions. In summary, these results imply that the number of CD8Teff_3 is increased after MTT treatment compared to RFA treatment, which can exert strong T-cell activation function, thus possibly contributing to the effector function. It also suggests that the superior prognosis of MTT treatment over RFA treatment may be attributed to the proliferation of CD8Teff_3 after MTT treatment that can activate T-cells.

**Figure 7:**
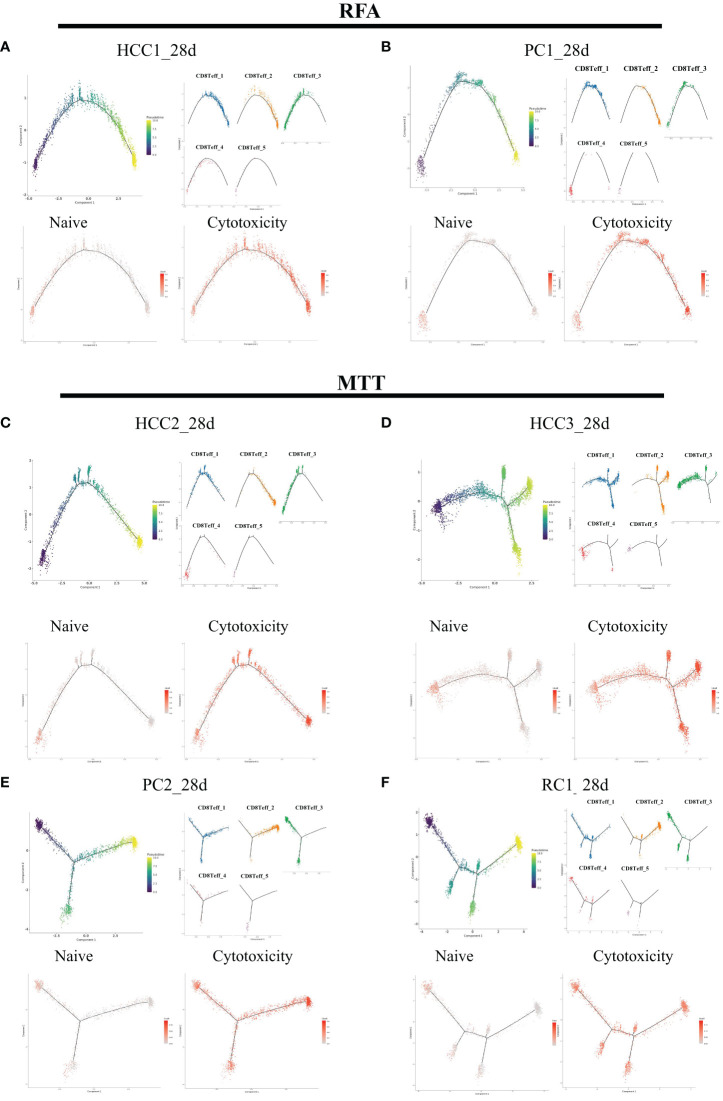
CD8Teff cells on 28 days were analyzed in pseudo-time. The five clusters of C8Teff as well as naive and cytotoxic gene set Ucell scores were mapped on the pseudo-time axis. **(A, B)** Trajectory analysis results of two samples in RFA group. **(C–F)** Trajectory analysis result of four samples in MTT group.

To verify that CD8Teff_3 is an important source of CD8^+^ Teff cells activation, pseudotime analysis was performed on CD8^+^ Teff cells on day 28 (**Figure 7**). In addition, UCell scoring was conducted using Naïve gene sets (*CCR7, TCF7, LEF1, SELL*) and cytotoxic gene sets (KLRG1, KLRD1, GZMB, GZMA, GMLY, PRF1, GZMM, NKG7, TBX21, ZEB2, HOPX, GZMH, KLRK1, IFNG, CCL3, CST7, ADGRG1, IL32, CRTAM, CX3CR1, KLRC1, FGFBP2, FCGR3A, NCR3, CCL4). The scoring results were mapped on the pseudotime axis. The results showed a gradual decrease in Naive score of CD8^+^ Teff cells along pseudotime axis in both MTT and RFA groups, while cytotoxicity score showed a gradual increase. By mapping the cell subsets on the pseudotime axis, it was observed that the starting state of both MTT and RFA groups was composed of cluster 4 and part of cluster 3 cells. This finding suggested that the cluster 4 and part of cluster 3 cells may be the mother cell of CD8^+^ Teff cells differentiation. Cluster 1, on the other hand, was located in the middle of the pseudotime axis, and cluster 2 was at the end. These results are consistent with the previous Jarcard similarity analysis. Hence, it demonstrates that CD8Teff_3 may be the mother cell for CD8^+^ Teff cells differentiation, and MTT treatment relies on CD8Teff_3 to activate CD8^+^ Teff cells, driving CD8^+^ Teff cells to perform its effector function.

## 4 Discussion

Previous studies have shown that MTT treatment can help improve the therapeutic effect of CRCLM patients by promoting CD4^+^ T cell-mediated antitumor responses and reducing regulatory T cells (10). The application of single-cell sequencing in cancer research has transformed our understanding of the biological features and dynamics of cancer lesions, which is very helpful in tumor diagnosis, targeted therapy, and prognosis prediction (27). Therefore, this technology was applied in our study to further investigate the mechanism responsible for the superior prognosis of MTT over RFA treatment. It also explained how MTT treatment regulates T cells to perform good immune functions. In this study, single-cell sequencing was employed to analyze the peripheral blood immune landscape in patients with liver, pancreatic, and colorectal cancers who underwent MTT and RFA treatment. A total of 9 types of immune cells were annotated, among which the T cells and ClassicalMo were the most dominant. Both treatments had an impact on the composition of peripheral blood immune cells, and the difference was not significant. However, compared to RFA treatment, MTT treatment showed an increasing trend in T cells. Cancer develops in complex tissue environments and relies on these environments to sustain growth, invasion, and metastasis (28). In addition, the immune cells infiltrating the TME not only play a role in tumor growth and progression but also have an important impact on the outcome of disease treatment and patient prognosis. T cells have been reported to effectively resist tumor growth by exerting their immune functions such as cytotoxicity and killing in the tumor microenvironment (TME). As aforementioned, an increasing trend in T cells after MTT treatment was found by inter-group comparison of the changes in the proportion of immune cells. However, despite the high proportion of ClassicalMo cells in both groups, there were significant differences in antigen presentation and other related immune functions only on day 3, but not on day 7 and 28. Based on the study background and results above, we focused on the in-depth research on the differences in immune functions of T cells between MTT and RFA and explored the key reasons why MTT has a better prognosis than RFA.

Radiofrequency ablation achieves the therapeutic effect by heating up local tumor lesions. Damage-associated molecular patterns (DAMPs) from necrotic tissues further affect the TME, including RNA and DNA (29). Zerbini et al. found that cellular fragments released from tumor tissue after RFA promoted the activation of the immune system (30) and induced an increase in plasma levels of IL-1, IL-6, IL-8, and TNF-a (31). Another tumor ablation treatment, cryoablation, uses very cold temperatures to freeze and kill tumor cells (32, 33). The local tumor progression rate of liver malignancies (>3cm) after cryoablation was significantly lower than RFA treatment (34). In addition, cryoablation can produce an “abscopal effect” (35), i.e., the immune response stimulated by the immunoactive antigen derived from the tumor tissue may help treat metastatic cancer at distant sites (36). In terms of promoting immune system, the ultra-low temperature of cryoablation can induce tissue destruction (37) and releases DAMPs, including DNA, tumor antigens, cytokines, and inflammatory factors (38). Therefore, both MTT and RFA therapies can affect the immune function of the patients. So, what are the differences in the immune function characteristics in T cells between the two treatments? First, DEGs and transcription factor analysis have shown that T cell activation was higher in MTT-treated patients than in RFA-treated patients, and T cells may be undergoing effective proliferation processes. In order to investigate whether there is a certain T cell subset that plays a major role in immune function, T cells were further subdivided into 6 subsets. Then, in clustering analysis of expression pattern on the 6 T cell subsets, and clusters with an increasing trend for functional enrichment analysis were confirmed. It was found that both treatments could effectively activate pro-inflammatory and other signaling pathways in peripheral blood. However, the inter-group difference analysis revealed that these pathways were more significantly upregulated after MTT treatment and could exert better immune functions. Surprisingly, after RFA treatment, all T cell subsets were more enriched in the RAGE signaling pathway. The RAGE signaling pathway can promote the differentiation of CD4^+^ T cells into Th17 cells, which may break the balance between Th1 and Th2, leading to the worsening of the pathological state or adverse prognosis in the later stages (39). Based on previous studies, MTT treatment has been shown to promote CD4^+^ T cells and reduce regulatory T cells, leading to anti-tumor effects. On the other hand, the increased enrichment of the RAGE signaling pathway after RFA treatment may be one of the important reasons for its poor prognosis. The upregulation of pro-inflammatory signals such as TNF-α, IFN-α, and TGF-β was more significant after MTT treatment, which enables MTT to exert a stronger immune response and improve the poor prognosis of liver malignancies patients.

By analyzing the pathway expression of CD8^+^ Teff cells after the two treatments, it is suggested that MTT not only affects T cell activation in the TME but also indirectly activates T cells in peripheral blood. Meanwhile, pro-inflammatory signals such as IFN-γ and IFN-α in CD8^+^ Teff cells were always significantly upregulated within 3-28 days after MTT treatment, indicating that MTT may utilize these signaling pathways to release more effectors to exert anti-tumor effects. Our previous study revealed that IFN-γ expression was lower in the MTT group than in the RFA group in 3 months after treatment, but IFN-γ expression was upregulated in CD8^+^ Teff cells in the MTT group in 6 months (10). This further demonstrates that MTT can activate peripheral CD8^+^ Teff cells more effectively than RFA. In addition, the PI3K-AKT-mTOR signaling pathway was significantly upregulated in CD8^+^ Teff cells after MTT treatment on day 28, and the expression of PIK3R1 increased over time. The PI3K-AKT-mTOR signaling pathway has been reported to be critical in T cell activation and function (40), and PI3K activation in T cells promotes survival and cell cycle progression, regulating their differentiation (41). The expression downregulation of apoptosis-related genes of *S100A11*, *GIMAP7*, and *GIMAP4* in CD8^+^ Teff cells after MTT compared with RFA treatment was also verified in this study. It further confirmed that, in addition to enhancing the expression of inflammatory signals, MTT may also rely on the PI3K-AKT-mTOR pathway to promote the survival of CD8^+^ Teff cells. The subsequent exploration of CD8^+^ Teff cells revealed that the CD8Teff_3 subset, possessing a noteworthy capacity for T-cell activation function, may serve as the primary origin of CD8^+^ Teff cells, and the proportion of CD8Teff_3 cells was higher in MTT than in RFA treatment. This may promote the activation and differentiation of CD8Teff_3 activation, reduce the immunosuppressive functions in the peripheral blood environment, and play a role in remodeling the peripheral blood environment. Also, considering technologies such as RFA and cryoablation have local efficacy and they can not directly lead to apoptosis in peripheral blood, we presume that the activation of T cells and immune effector functions in MTT treatment may also depend on the activation of antigen presentation. The antigen-binding signaling pathway was significantly upregulated in CD8^+^ Teff cells within 3 to 28 days after MTT compared to RFA treatment, and significantly increased on day 28 compared to day 0. This may indicate that MTT treatment could also activate DAMPs activation in tumor cells and recruit peripheral T-cell activation and proliferation, thus effectively promoting therapeutic efficacy and contributing to a certain “abscopal effect” on distant metastases. It is worth noting that the current understanding of the effects of RFA or cryoablation on T cells mostly focuses on activation or functional reprogramming. However, in this study, potential metabolic showed differences in peripheral T cells after MTT and RFA treatment. Specifically, in MTT group, pathways such as MYC target, hypoxia, and glycolysis were upregulated after treatment, which are crucial for T cell activation. The metabolic program of activated T cells in a resting state is similar to the metabolic shift that occurs in tumors, involving a series of transitions from catabolic metabolism to widespread anabolic metabolism processes (42). Even in the presence of adequate oxygen supply, tumorigenic signals can induce aerobic glycolysis *via* PI3K and MYC pathways, and similarly, activation of TCR by costimulatory signals will induce and limit the extent of inflammatory response and proliferation of T cells (42). To support the evolving metabolic demands, T cells upregulate nutrient transport proteins and increase the uptake of glucose and amino acid (42). After T cell activation, under the influence of inflammatory and cytokine stimuli, different metabolic programs are induced, leading to different effector functions (42). CD8^+^ T cells tend to rely on glycolysis for the production of IFN-γ. During IFN-γ production, CD8^+^ T cells tend towards glycolytic pathway (43), while the transcriptional activities of MYC and hypoxia-inducible factor 1 (HIF-1) are upregulated upon T cell activation and promote metabolic reprogramming (44). These two factors activate the expression of genes encoding enzymes that promote glycolysis (e.g. pyruvate kinase M1 (PKM1), hexokinase 2 (HK2), and GLUT1), and the pathway from proximal metabolites in the glycolysis pathway are also players in T cell activation and function (45). In addition, lactate dehydrogenase A (LDHA) in glycolysis is induced by PI3K signaling in CD8^+^ Teff cells enhancing T cell immune activity (46). We will further explore the metabolic reprogramming in CD8^+^ Teff cells after MTT treatment.

In summary, by using single-cell sequencing, we compared the immune environment in peripheral blood of liver malignancies patients receiving MTT and RFA treatment. The results showed that 1) both treatments can effectively activate CD8^+^ Teff cells in the peripheral blood, but MTT has a stronger promoting effect on cell anti-tumor immune activity than RFA; 2) MTT treatment activates the PI3K-AKT-mTOR pathway and significantly upregulates the response to interferon stimulation in CD8^+^ Teff cells; 3) compared with RFA, MTT treatment can more effectively induce CD8^+^ Teff cells to exert the effect function, which helps both cells to play synergistic roll tumor immunotherapy. These findings elucidate the mechanism behind the better prognosis of MTT treatment compared to RFA treatment and provide the theoretical basis for the clinical application of MTT treatment. In particular, the potential effects of MTT on T cells metabolism and synergistic interaction between immune cells will be the focus of our future studies.

## Data availability statement

The raw sequence data reported in this paper have been deposited in the Genome Sequence Archive in National Genomics Data Center (Beijing, China) under the BioProject ID PRJCA016914 that are publicly accessible at https://ngdc.cncb.ac.cn/gsa-human.

## Ethics statement

The studies involving human participants were reviewed and approved by Medical Ethics Committee of Fudan University Cancer Hospital. Written informed consent to participate in this study was provided by the participants’ legal guardian/next of kin.

## Author contributions

W-TL, L-CX and LX contributed to the conception and design of the study. YW, G-ZW, CC, H-ZH and X-HH completes the surgery, sample collection, and experimental parts. YW and G-ZW performed the statistical analysis. YW and G-ZW wrote the first draft of the manuscript. CC, H-ZH, Y-HW and X-HH wrote sections of the manuscript. All authors contributed to the article and approved the submitted version.

## Supplementary material

Supplementary Figure 1 GSEA analysis of classical monocyte Antigen Delivery Pathway (3d, 7d, 28d).

Supplementary Figure 2 Clustering and enrichment analysis of expression patterns in T cell subsets in MTT treatment group. **(A)**: expression pattern fitting of CD8^+^ Naive T cells in MTT group. **(B)**: GO enrichment analysis was conducted for CD8^+^ Naive T fitting gene set cluster6 in MTT group. **(C)**: expression pattern fitting of Treg in MTT group. **(D)**: GO enrichment analysis was conducted for Treg fitting gene set cluster 5 in MTT group. **(E)**: expression pattern fitting of γδT cells in MTT group. **(F)**: GO enrichment analysis was conducted for γδT cells fitting gene set cluster 2 in MTT group. **(G)**: expression pattern fitting of Proliferation T cells in MTT group. **(H)**: GO enrichment analysis was conducted for Proliferation T cells fitting gene set cluster 1 in MTT group.

Supplementary Figure 3 Clustering and enrichment analysis of expression patterns in T cell subsets in RFA treatment group. **(A)**: expression pattern fitting of CD8^+^ Naive T cells in RFA group. **(B)**: GO enrichment analysis was conducted for CD8^+^ Naive T fitting gene set cluster6 in RFA group. **(C)**: expression pattern fitting of Treg in RFA group. **(D)**: GO enrichment analysis was conducted for Treg fitting gene set cluster 5 in RFA group. **(E)**: expression pattern fitting of γδT cells in RFA group. **(F)**: GO enrichment analysis was conducted for γδT cells fitting gene set cluster 2 in RFA group. **(G)**: expression pattern fitting of Proliferation T cells in RFA group. **(H)**: GO enrichment analysis was conducted for Proliferation T cells fitting gene set cluster 1 in RFA group.
