# Supplementary figures and images for: Exploration of the impact of multimode thermal therapy versus radiofrequency ablation on CD8+ T effector cells of liver malignancies based on single cell transcriptomics
Source: Front Immunol. 2023 Jun 2;14:1172362. doi: 10.3389/fimmu.2023.1172362 (PMC10272448; doi:10.3389/fimmu.2023.1172362)

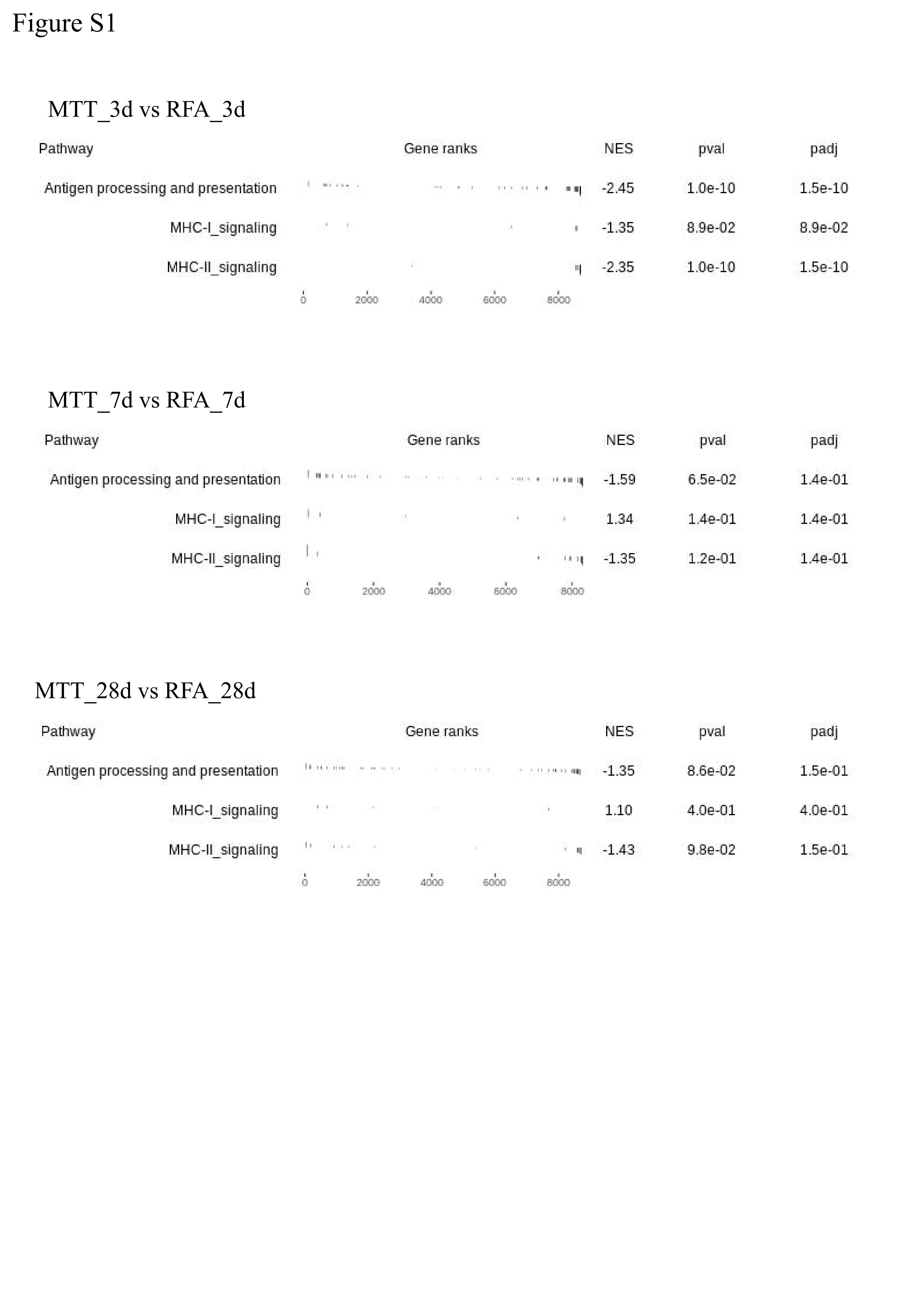

Supplement: Supplementary Figure 1 — GSEA analysis of classical monocyte Antigen Delivery Pathway (3d, 7d, 28d). [file Image_1.jpeg]

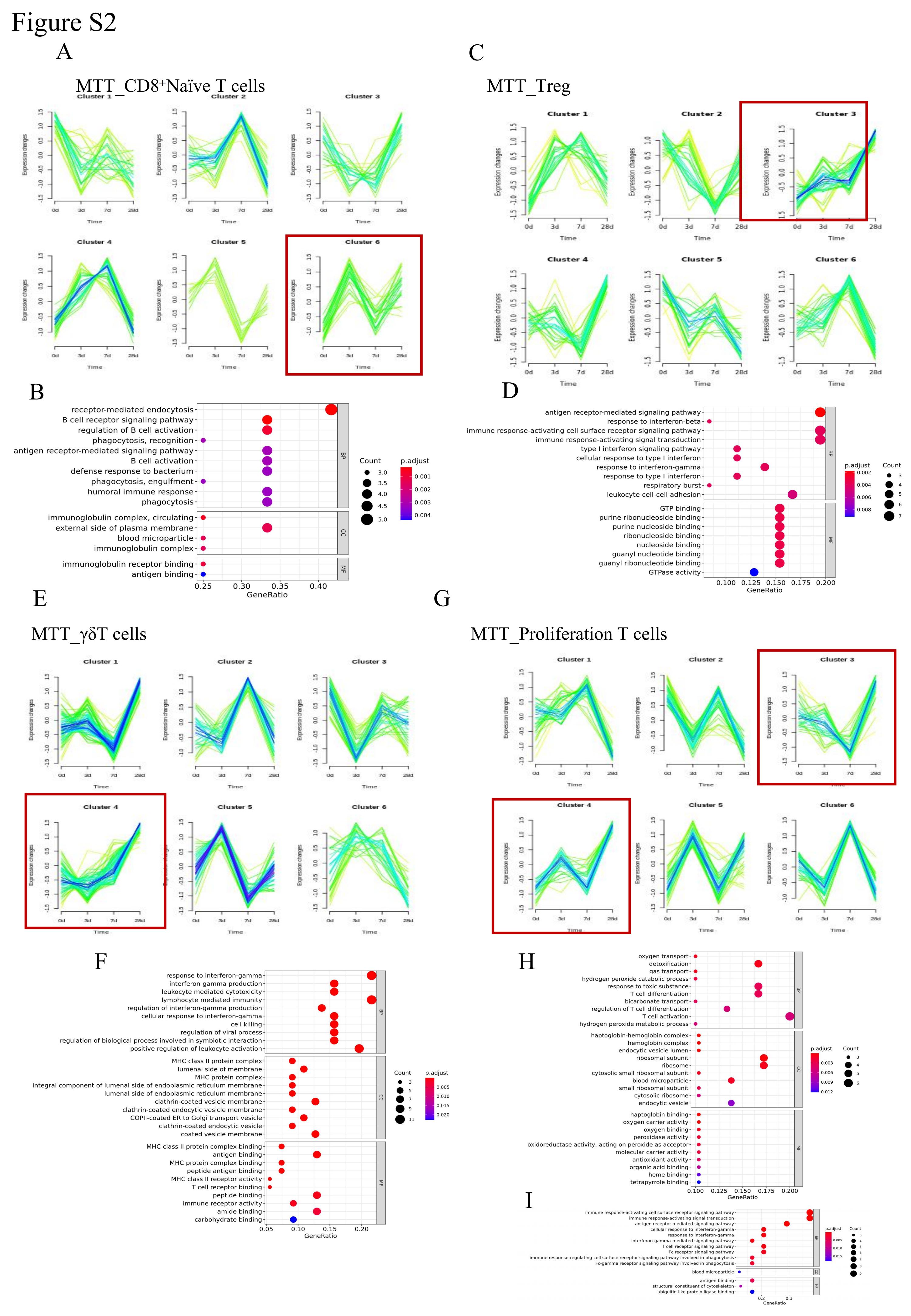

Supplement: Supplementary Figure 2 — Clustering and enrichment analysis of expression patterns in T cell subsets in MTT treatment group. (A): expression pattern fitting of CD8+ Naive T cells in MTT group. (B): GO enrichment analysis was conducted for CD8+ Naive T fitting gene set cluster6 in MTT group. (C): expression pattern fitting of Treg in MTT group. (D): GO enrichment analysis was conducted for Treg fitting gene set cluster 5 in MTT group. (E): expression pattern fitting of γδT cells in MTT group. (F): GO enrichment analysis was conducted for γδT cells fitting gene set cluster 2 in MTT group. (G): expression pattern fitting of Proliferation T cells in MTT group. (H): GO enrichment analysis was conducted for Proliferation T cells fitting gene set cluster 1 in MTT group. [file Image_2.jpeg]

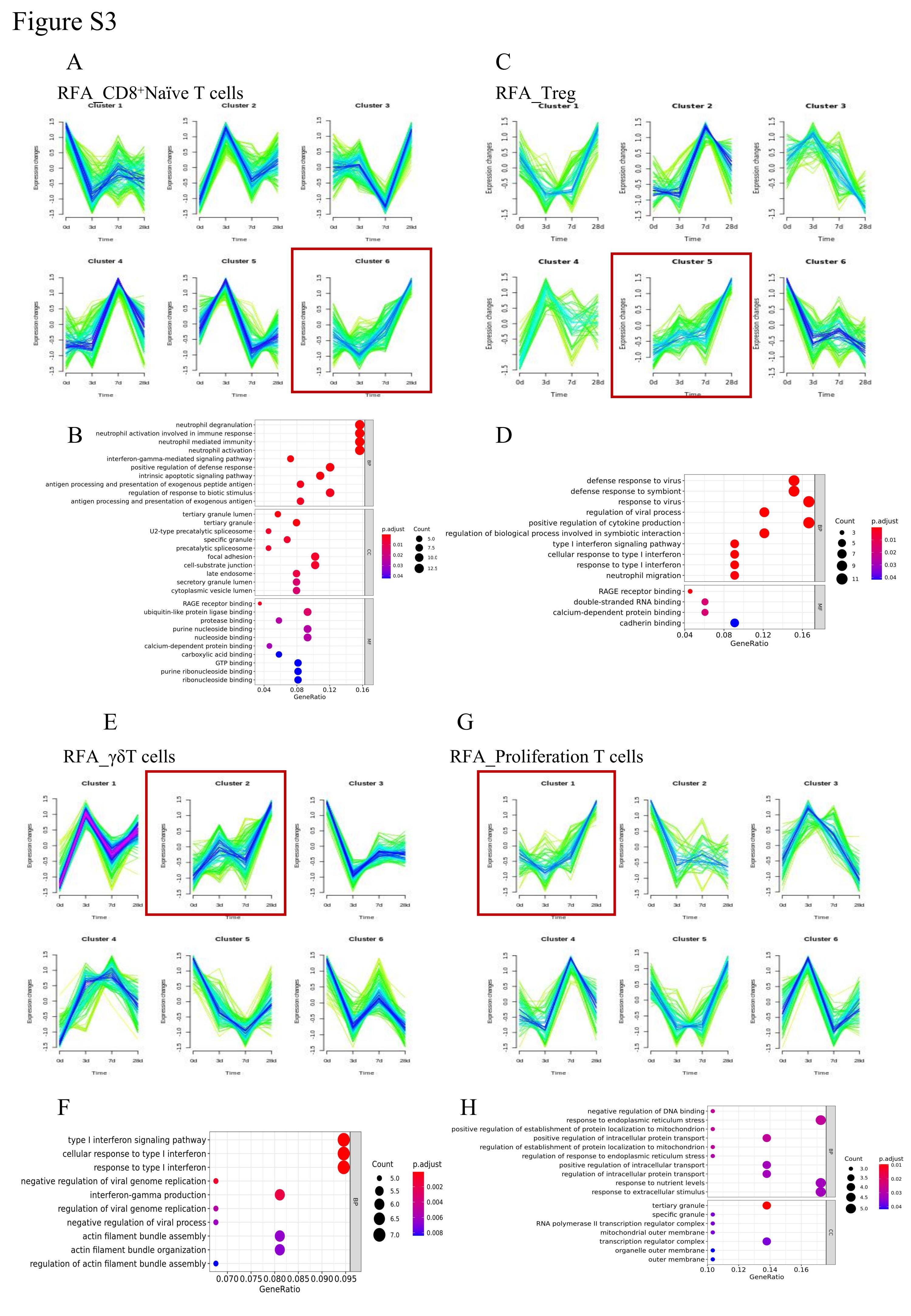

Supplement: Supplementary Figure 3 — Clustering and enrichment analysis of expression patterns in T cell subsets in RFA treatment group. (A): expression pattern fitting of CD8+ Naive T cells in RFA group. (B): GO enrichment analysis was conducted for CD8+ Naive T fitting gene set cluster6 in RFA group. (C): expression pattern fitting of Treg in RFA group. (D): GO enrichment analysis was conducted for Treg fitting gene set cluster 5 in RFA group. (E): expression pattern fitting of γδT cells in RFA group. (F): GO enrichment analysis was conducted for γδT cells fitting gene set cluster 2 in RFA group. (G): expression pattern fitting of Proliferation T cells in RFA group. (H): GO enrichment analysis was conducted for Proliferation T cells fitting gene set cluster 1 in RFA group. [file Image_3.jpeg]
